# *DDX41* germline variants causing donor cell leukemia indicate a need for further genetic workup in the context of hematopoietic stem cell transplantation

**DOI:** 10.1038/s41408-023-00846-2

**Published:** 2023-05-10

**Authors:** Benjamin Rolles, Robert Meyer, Matthias Begemann, Miriam Elbracht, Edgar Jost, Matthias Stelljes, Ingo Kurth, Tim H. Brümmendorf, Gerda Silling

**Affiliations:** 1 Department of Hematology, Oncology, Hemostaseology and Stem Cell Transplantation, Medical Faculty, RWTH Aachen University, Aachen, Germany; 2 Center for Integrated Oncology Aachen Bonn Cologne Duesseldorf (CIO ABCD), Aachen, Germany; 3 Division of Hematology, Department of Medicine, Brigham and Women’s Hospital, Harvard Medical School, Boston, MA USA; 4 Institute for Human Genetics and Genomic Medicine, Medical Faculty, RWTH Aachen University, Aachen, Germany; 5 Department of Medicine A, Hematology and Oncology, University of Muenster, Münster, Germany

**Keywords:** Acute myeloid leukaemia, Risk factors

Hematopoietic stem cell transplantation (HSCT) represents the standard treatment for patients with high or intermediate-2 risk (IPSS classification) myelodysplastic syndrome (MDS) and acute myeloid leukemia (AML) with intermediate or unfavorable risk (ELN classification) [1]. Despite recent progress in both induction treatment as well as conditioning regimens before transplantation and immunosuppression thereafter, relapse of MDS and AML occurs in 30–50% of patients and remains the most frequent cause of mortality. Disease relapse originates in almost all cases from residual leukemic stem cells escaping the chemotherapeutic and (allo-) immunological effects of transplantation. Rarely “relapse” actually originates from donor-derived cells. These so-called donor cell leukemias (DCL) reflect approximately 2% of AML or MDS relapses after HSCT [1].

Following the exclusion of relevant underlying hematological and non-hematological disorders, excluding their suitability to donate stem cells, donors are being checked for matches in established HLA loci and compatibility with the recipient. However, genetic screening of the patient and, particularly, the stem cell donor for inherited predispositions towards hematopoietic stem cell disorders such as diseases like MDS or AML is currently not part of the routine clinical work up in most cancer centers, especially not in adult patients.

We report a male Caucasian patient who received his second allogeneic HSCT at the University Hospital RWTH Aachen at the age of 69 years after having developed DCL (Fig. 1A). At the age of 59 years, he had first been diagnosed with a progressive MDS and excess of blasts (EB2) accompanied by neutropenia, thrombocytopenia as well as multiple infectious complications. Following conditioning with Fludarabine and Busulfan, the patient was transplanted from his HLA-identical brother without induction treatment before HSCT. Despite timely engraftment and full donor cell chimerism, the peripheral blood cell count showed mild leukopenia and thrombocytopenia (Common Terminology Criteria for Adverse Events (CTCAE) grade 2–3) during the post-transplant follow-up. Ten years after the first HSCT, a further decrease in platelet count from grade 3 to grade 4 was observed, and the blood smear showed 1% blasts. Bone marrow work-up revealed an AML with 22% blasts, normal karyotype, and a donor cell chimerism of 100%. Thereby, DCL was diagnosed. The molecular work-up led to the detection of a *DNMT3A* variant with a variant allele frequency (VAF) of 12% (c.2249 C > G, p.(Pro750Arg)). While the retrospective analysis of the patient’s bone marrow before the first HSCT did not reveal the described *DNMT3A* variant, workup of the donor’s blood at the time of donation led to the confirmation of the *DNMT3A* variant with similar VAF compared to the patient’s blood after HSCT demonstrating transplanted clonal hematopoiesis. After re-induction chemotherapy with daunorubicin and cytosine-arabinoside (7 + 3), complete remission of the DCL was achieved, and following conditioning with fludarabin, treosulfan and antithymocyte globuline (ATG), a second HSCT from a matched unrelated donor (with 6.7 × 10^6^ CD34^+^ cells/kg BW) was performed. Myeloid engraftment occurred on day 23 with >500 G/l granulocytes and on day 29 with >20 G/l platelets together with complete donor cell chimerism. The patient developed a grade I acute graft-versus-host disease (GvHD) of the skin treated topically by corticosteroids. At the time of this report, the patient is now 73 years old and in complete remission of his DCL 42 months after HSCT without significant signs of chronic GvHD.

**Fig. 1:**
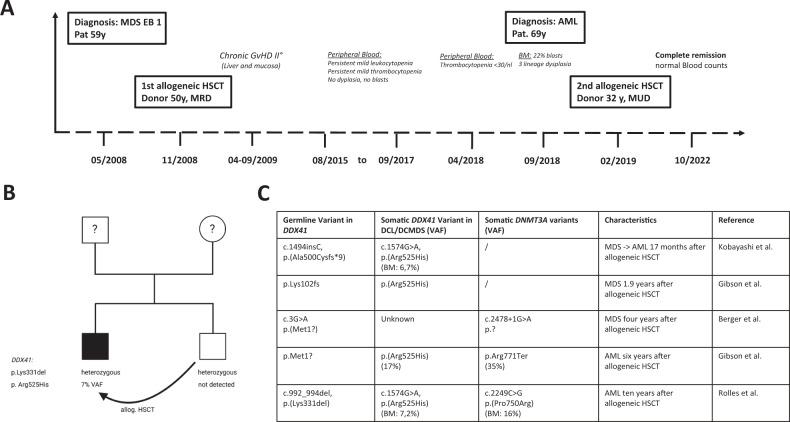
*DDX41*-positive donor cell leukemia. **A** Timeline of disease course of the reported male patient with donor cell leukemia (DCL). **B** Pedigree of the family of our patient with DCL. **C** Detected germline and somatic variants in *DDX41* as well as *DNMT3A* mutational status after diagnosis of DCL of our case and reported cases of *DDX41*-associated DCL from the literature. *Stated as p.F498fs in the original article. AML acute myeloid leukemia, BM bone marrow, BW body weight, DCL donor cell leukemia, DCMDS donor cell myelodysplastic syndrome, HSCT hematopoietic stem cell transplantation, MDS myelodysplastic syndrome, VAF variant allel frequency, DDX41 NM_016222.4, DNMT3A NM_022552.5.

A genetic workup of the patient and his related donor was performed after informed consent for genetic germline testing. The study was approved by the ethics committee of the University Hospital RWTH Aachen, Germany (EK 206/09) and was conducted in accordance with the ethical standards due to the Declaration of Helsinki. The patient had no other siblings except his brother. No tumor predispositions were known in the family.

Whole exome sequencing was performed using DNA samples isolated from the hair roots of the patient (germline) and a blood sample taken at the time of DCL diagnosis (tumor). An underlying heterozygeous 3-bp deletion (c.992_994; p.(Lys331del)) was identified in the Dead-Box Helicase 41 (*DDX41*) gene in the germline of the patient. Subsequent analysis of the brother’s hair roots and blood using targeted Sanger sequencing confirmed the germline status of the *DDX41* variant in the heterozygous state. At the time of manuscript preparation, the brother did not show any clinical abnormalities that could indicate the presence of the predisposition.

There is increasing evidence that inherited changes of *DDX41* predispose to late-onset MDS and AML [2, 3]. Although *DDX41* is located on chromosome 5q35, the detection of variants in myeloid diseases is overrepresented in male patients suggesting an impact of gender on the penetrance [3]. Interestingly, in 1690 screened AML patients, 5% carried *DDX41* germline variants, and 82% of them showed further *DDX41* variants as a second somatic aberration supporting the assumption that *DDX41* loss of function is a main driver of AML [4]. *DDX41*-positive AML was previously clinically characterized by a low amount of bone marrow blasts and normal karyotype [4, 5], well in line with the findings in the patient reported here. In the context of germline aberrations in *DDX41*, a high number of unreported cases is assumed due to the low penetrance and late age of disease onset. Interestingly, AML patients with *DDX41* variants show a better response to induction and consolidation chemotherapy, a lower risk of 1-year-disease relapse, and prolonged relapse-free survival after HSCT [4]. To the best of our knowledge, there are three case reports showing DCL in the context of a causative *DDX41* germline variant so far [6–8].

The germline variant identified in our patient has previously been observed in MDS/AML patients [2, 9]. Genetic workup of the DCL bone marrow sample revealed the somatic appearance of a second variant of the *DDX41* gene (c.1574 G > A, p.(Arg525His)) with a variant allele frequency of 7.2% (Fig. 1B). This variant has been described as a recurrent “second somatic hit” in the pathogenesis of *DDX41*-associated MDS/AML and once in the pathogenesis of DCL [7]. We report a family that shows the involvement of *DDX41* in the context of donor cell leukemia as one of the few known cases [6–8]. Furthermore, we point out the high clinical importance of the *DDX41* data published by Berger et al. [6], Kobayashi et al. [7], and Gibson et al. [8] (Fig. 1C). Detecting recurrent somatic *DDX41*-variants as c.1574 G > A, p.(Arg525His) supports the assumption that *DDX41* is a strong driver of DCL.

Based on previous publications and our new data, concomitant variants in *DNMT3A* seem to be frequent in the context of *DDX41*-driven DCL. Given that Gibson et al. found a positive correlation between *DNMT3A* variants on relapse-free survival after HSCT in general [8], this may explain why the indicated case of DCL arose after a long latency of almost 10 years. Consistent with previous publications, we do not believe that the concomitant *DNMT3A* variant had a causal (negative) impact on DCL pathogenesis [8, 10].

Although DCL is an extremely rare event and only 5% of AML patients carry a *DDX41* germline variant, the number of patients identified with a *DDX41* variant in the context of DCLs is increasing due to comprehensive pathogenetic analyses. Subsequently, it can be assumed that *DDX41* germline variants are frequently involved in the development of DCL. Interestingly, little is known about the pathogenesis of AML driven by *DDX41* variants. Due to the fact that telomere shortening was shown in patients harboring *DDX41* germline variants [11], and telomere attrition was demonstrated in donor cells after HSCT [12], involvement of telomere biology in progenitor and stem cells can be speculated in the context of DCL pathogenesis.

With the currently applied standard screening tests for patients with AML/MDS and donors, predisposing genetic variants are often not identified. However, knowledge about them is important to avoid cell transplantation from a family donor who carries a pathogenic variant leading to an increased risk of developing DCL. As there is no central registry for family donors in Germany, it would be reasonable to gather data on these healthy donors and their medical history related to molecular findings in a central registry for HSCT to improve HSCT follow-up statistics.

Based on recent data, we suggest genetic screening of leukemia and MDS patients as well as their related stem cell donors for variants in disease-predisposition genes. Knowledge about the genetic risk profile impacts clinical decision-making and HSCT donor selection, even if the stem cell donor presents healthy at the time point of transplantation.

## Data Availability

Upon a reasonable request, the data presented will be made available.
